# Beyond Genes: Germline Disruption in the Etiology of Autism Spectrum Disorders

**DOI:** 10.1007/s10803-021-05304-1

**Published:** 2021-10-01

**Authors:** Jill Escher, Wei Yan, Emilie F. Rissman, Hsiao-Lin V. Wang, Arturo Hernandez, Victor G. Corces

**Affiliations:** 1 Escher Fund for Autism, 1590 Calaveras Avenue, San Jose, CA USA; 2 The Lundquist Institute for Biomedical Innovation at Harbor-UCLA Medical Center, Torrance, CA USA; 3 Department of Medicine, David Geffen School of Medicine at UCLA, Los Angeles, CA USA; 4 Center for Human Health and the Environment and Department of Biological Sciences, North Carolina State University, Raleigh, NC USA; 5 Department of Human Genetics, Emory University School of Medicine, Atlanta, GA USA; 6 Maine Medical Center Research Institute, MaineHealth, Scarborough, ME USA; 7 Tufts University School of Medicine, Boston, MA USA

**Keywords:** Germ cells, Epigenetics, Genetic toxicology, Non-genetic inheritance, Autism spectrum disorder, Gene expression

## Abstract

Investigations into the etiology of autism spectrum disorders have been largely confined to two realms: variations in DNA sequence and somatic developmental exposures. Here we suggest a third route—disruption of the germline epigenome induced by exogenous toxicants during a parent’s gamete development. Similar to cases of germline mutation, these molecular perturbations may produce dysregulated transcription of brain-related genes during fetal and early development, resulting in abnormal neurobehavioral phenotypes in offspring. Many types of exposures may have these impacts, and here we discuss examples of anesthetic gases, tobacco components, synthetic steroids, and valproic acid. Alterations in parental germline could help explain some unsolved phenomena of autism, including increased prevalence, missing heritability, skewed sex ratio, and heterogeneity of neurobiology and behavior.

## Introduction

**Fig. 1:**
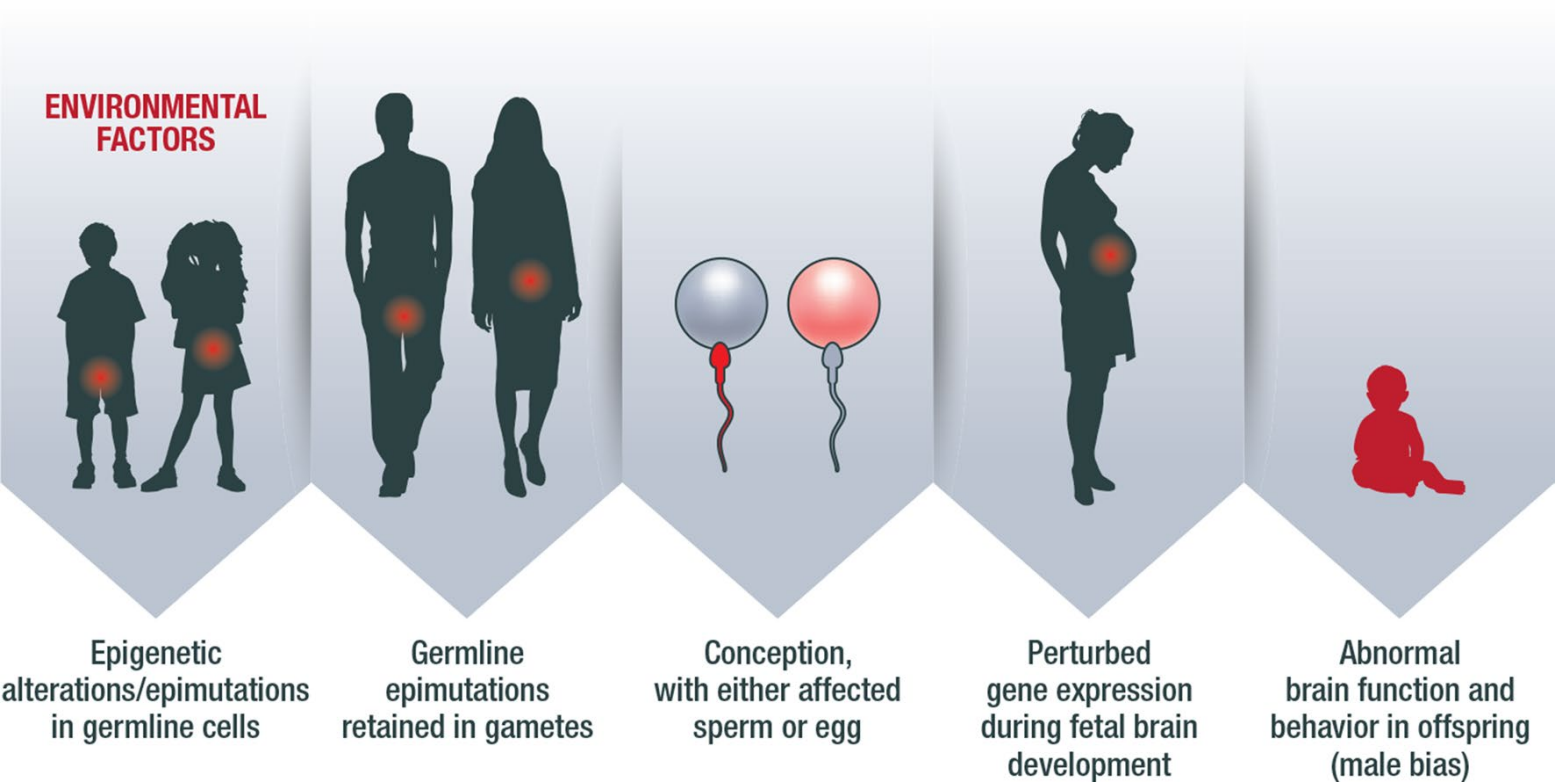
Conceptual Diagram of Non-Genetic Inheritance of Autism. The diagram illustrates a general overview of how non-genetic inheritance might occur in a case of autism. As an example, an exposure to a toxicant (e.g., anesthetic gas or EDC, as discussed in this paper) could cause epimutation in the germline (in this example, the pool of spermatogonial stem cells in the male, or the pool of oocytes in the female), at genes related to brain development. This then leads to DNA methylation abnormalities in the mature sperm or ovum. Upon the conception, the pattern is retained, perturbing gene expression and the normal process of brain development in the fetus and young child. The offspring exhibits a phenotype of abnormal neurodevelopment and behaviors. This figure features childhood exposures as an example, but a similar phenomenon may occur in other periods as well

In the domain of autism spectrum disorder (ASD, or autism) and neurodevelopmental pathology research, it is commonly assumed that the etiological processes of concern are limited to variations in DNA sequence or somatic environmental insults (Dietz et al., 2020; Lord et al., 2020). In this commentary, we propose that this binary approach—“genetics” or “environment”—is incomplete as it overlooks another critical dimension of risk for neurodevelopmental disorders. Specifically, we suggest that exogenously induced germ cell perturbations, including but not limited to disruptions of the transcriptional machinery, chromatin structure/organization, or other epigenomic information such as non-coding RNAs, contribute to the heritable risk for neurodevelopmental pathology, which may manifest as autism or related disorders (Fig. 1).

To be clear, this “germline disruption” hypothesis is not addressing what is often referred to as “genes by environment,” or the possibility that environmental factors influence gene expression during the course of fetal or early development, which is best understood as a subcategory of somatic exposure. Rather, we focus on exposures to, and perturbations of, the germline of the affected individual’s parent(s) over the course of the parental gametogenesis. These insults to the germline may occur at various life stages, beginning when primordial germ cells are initially formed in the parents during embryonic life, continuing through puberty to the time of conception of the next generation, thus providing a prolonged opportunity for multiple types of exposures to disrupt transcriptional, epigenetic, or genetic programs (Fig. 1).

### The Missing Heritability of ASD

Our proposal goes straight to the question of the missing heritability of ASD. ASD is a broad category term that encompasses neurodevelopmental disorders of mostly unknown etiology that are characterized by impairments in social-communication, sensory dysfunction, the presence of restrictive interests and repetitive behaviors, and varying degrees of intellectual and adaptive disability (Lord et al., 2020). While there is no single pathophysiology of autism, studies point to dysregulation of genes during fetal and early life brain development, affecting cell proliferation, differentiation, neurogenesis, and migration, resulting in atypical patterning in the cerebral cortex, among other pathologies (Courchesne et al., 2019). Perturbed transcriptional pathways are seen broadly in autism cases, both in studies based on peripheral tissues and post-mortem autism brains (Gazestani et al., 2019; Gupta et al., 2014; Quesnel-Vallières et al., 2019; Voineagu et al., 2011). The transcriptional dysregulation of autism seems to occur notwithstanding the absence of any detected genetic mutations.

Epidemiological studies have yielded puzzling findings. In what seems like an irreconcilable paradox, the prevalence of autism has been steadily increasing (Boyle et al., 2011; Maenner et al., 2020; Nevison, 2014; Schendel & Thorsteinsson, 2018), reaching an estimated 1 in 54 U.S. children according to the Centers for Disease Control (Maenner et al., 2020), while at the same time studies repeatedly demonstrate its strong heritability. This heritability, generally seen to be approximately 50–80%, has been observed in twin studies (Castelbaum et al., 2020; Sandin et al., 2017; Tick et al., 2016), in large national cohorts (Colvert et al., 2015; Sandin et al., 2014), and across populations (Bai et al., 2019). In contrast, there is generally weak evidence for prenatal or “maternal” factors broadly influencing risk (Bai et al., 2019), although certain environmental factors such as maternal valproate intake, maternal immune activation, and adverse perinatal events such as preterm birth and neonatal hypoxia can increase risk (Bölte et al., 2019; Lord et al., 2020). In addition, autism is also known to have a high recurrence risk among siblings, estimated to be 6.1% to 18.7% (Palmer et al., 2017).

Autism’s strong heritability and recurrence rate, in addition to its basis in transcriptional dysfunction in brain development, has led many in the field to presume the disorder must be genetic in origin, inspiring a “genetics first” research orientation (e.g., The Simons Vip Consortium, 2012) and a general assumption that a “genetic architecture” of autism will be identified (e.g., Pereanu et al., 2018). But there are many reasons for skepticism regarding the genetics-only view of ASD’s heritability.

First, despite massive investment in genetics research, genetic mutations are seen to contribute to only about 10–20% of cases, with most of this genetic risk stemming from de novo germline mutations (Lord et al., 2020; Pugsley et al., 2021), and with many, perhaps 10%, of the cases being rare syndromes involving multiple physiological and developmental pathologies extending beyond autism features (Zafeiriou et al., 2013). There is insufficient evidence to establish ASD specificity of any so-called “autism genes” (Myers et al., 2020), and potentially relevant genetic mutations, even when identified, often feature variable penetrance that may not result in an autism phenotype (De Rubeis & Buxbaum, 2015; Lord et al., 2020). Consistent with the observation that only about 10% of autism cases without syndromic features have a known genetic origin, a recent study conducted by the Simons Foundation for Autism Research Initiative identified genetic factors recognized to be causes or significant contributors to ASD in only 10.4% of cases that lacked a previous genetic diagnosis, while also identifying variants that are possibly associated with ASD in an additional 3.4% of families (Feliciano et al., 2019).

A role for common and normally harmless single-nucleotide polymorphisms acting additively is often speculated to exist, but despite much research regarding a possible role for common variants in explaining autism’s heritability, efforts to actually locate these variants have resulted in very weak explanatory power (Grove et al., 2019). A recent review and re-evaluation of genome-wide association studies found that almost no autism risk could be predicted accurately from single-nucleotide polymorphisms, and the authors cautioned that while twin studies tend to find strong heritability, unexamined epigenetic effects should be considered as playing a role (Patron et al., 2019). Furthermore, autism is associated with strong selective pressures against transmission, and reduced fecundity (Power et al., 2013; Pugsley et al., 2021), an expectation at odds with the steadily increasing rates of diagnosed autism.

Many studies have demonstrated an association between advanced paternal age and autism risk (Bölte et al., 2019), and to a lesser degree, advanced maternal age as well (Lord et al., 2020), both of which implicate aging germ cells. However, a father’s age-related germline mutations alone are unlikely to explain a significant portion of the increased risk for offspring autism (Gratten et al., 2016).

Studies using the full complement of genetic toolboxes have been unable to explain many other patterns seen in autism. While males are affected more often than females, at a ratio of about 4 to 1 (Fombonne, 2009; Maenner et al., 2020), a genetics-based explanation has not yet emerged. The high recurrence risk among siblings has yet to be explained by genetic studies. Notably, having a sibling with autism is the single strongest predictor of autism risk, stronger than any prenatal exposure or other known risk factor (Lord et al., 2020). Yet there is an unexplained genetic discordance among siblings who share an ASD diagnosis—in sibling pairs with ASD who carry rare autism-related mutations, fewer than one-third share those mutations (Yuen et al., 2015). Even in the relatively rare cases when a genetic diagnosis can be made, only a very small fraction of cases has implications for predicting risk in siblings (D’Abate et al., 2019).

In this commentary we offer a counterpoint to the conventional thinking about the roots of autism and suggest that the disorder’s etiology, heritability, sibling recurrence risk, increasing prevalence, neurobiology, heterogeneity and even its perplexing sex bias may at least in part be explained by the germline exposure history of parents and ancestors. This invokes the possibility that a toxicant-disturbed pool of parental germ cells (as opposed to, for example, harmless common genetic variants acting additively) should be the chief concern for autism causation research. To this end, we discuss (1) germ cell development and periods of vulnerability; (2) evidence for this germline disruption paradigm from both mammalian models and human studies; and (3) implications for future research.

## A Primer on Vulnerabilities of the Germ Cell Epigenome and Genome

Because autism research has largely lacked experience with the biological complexity and dynamism of human germline and its multifaceted heritable contents, we offer a brief primer on germ cell biology and how the associated epigenetic processes can result in an altered transcriptional landscape, leading to abnormal gene expression.

Prior to a successful conception and subsequent birth of a baby, germ cells undergo a lengthy process of gametogenesis to produce mature sperm or oocytes (Larose et al., 2019): depending on the age of the parents at conception, precursor cells to the mature sperm or egg could have spent anywhere from approximately 14 to 50 years in the gonads. While exogenous factors disrupting replication and recombination during meiosis may increase the risk for de novo mutagenesis (DeMarini, 2012), these factors can also perturb epigenetic and other non-genetic elements, including structural packaging of proteins, histone modifications, DNA methylation, coding and noncoding RNAs, and other cellular denizens such as mitochondria in the oocyte (Lempradl, 2020; Perera et al., 2020; Sales et al., 2017; Yeshurun & Hannan, 2019). Although toxicological paradigms tend to rely on the biological idea that “life starts at conception,” this is misleading from a molecular point of view, as the elements of the heritable program are dynamically synthesized in the gametes over many years before that moment.

The male and female germ cells initially develop in the same fashion. The diploid primordial germ cells (PGCs), the precursors of all germ cells, are first specified from the embryonic epiblast approximately 2–3 weeks after fertilization in humans. As the PGCs proliferate mitotically and migrate to the genital ridge, the precursor of the fetal gonads, they shed their former somatic epiblast identity as their DNA methylation is largely stripped away, from approximately 80% of the CpG dinucleotides methylated to approximately 5% in week nine (Tang et al., 2015). The period of low DNA methylation and reprogramming in the early germ cell may represent an exquisite window of susceptibility to toxicants due to the unprotected nature of the genomic DNA. While the nature of this susceptibility is not well understood, many toxicants such as endocrine disrupting chemicals (EDCs) are able to bind to various nuclear hormone receptors, which are transcription factors (TFs), and may elicit changes in transcription and occupancy of other TFs at enhancers and promoters (Martini et al., 2020). Following demethylation, the germ cells are remethylated in a sex-specific manner, as discussed below. Loss and acquisition of DNA methylation, including acquisition of genomic imprints that are specific to either sperm or egg, are necessary for the proper development of the future offspring, since abnormal methylation at imprinted genes can disturb gene expression, causing disorders such as neurodevelopmental impairment (SanMiguel & Bartolomei, 2018). For example, improper methylation of a single allele can result in imprinting disorders such as Prader–Willi Syndrome and Angelman Syndrome (Buiting, 2010). Another example is Rett syndrome, which is caused by deficiencies in MECP2 (an X-chromosome gene) and is associated with abnormal methylation in imprinted and autism candidate genes (Samaco et al., 2005). Apart from imprinted genes, TFs bound to specific sites in the sperm and oocyte genomes remain bound to the same sites in the embryo after fertilization (Jung et al., 2019). The altered patterns of TFs occupancy in germ cells could result in changes in gene expression when the embryo starts transcribing at the 2-cell stage.

### Considerations for Male Germline Development

The reprogramming of the male germline has been studied in greater detail than that of the female. In the male fetus, prospermatogonia undergo mitotic proliferation until entering mitotic arrest during the second trimester (Kurimoto & Saitou, 2019; Phillips et al., 2010). As directed by molecular signals coming from gonadal cells, male germ cells start DNA re-methylation by week 19 and this process is completed before birth (Wen & Tang, 2019). Shortly after birth, diploid prespermatogonia differentiate into type A spermatogonia, which resume mitosis at age 5–7 years and begin meiosis at puberty. Spermatogonial stem cells (SSCs) serve as the source for continuous production of mature sperm in adulthood (Neto et al., 2016). Damage to the genome or epigenome of SSCs, if unrepaired, will impact the quality of the final mature sperm derived from these cells. The SSCs are not exempt from external influence; rather, they communicate with the surrounding somatic cells (Sertoli cells) through gap junctions, endocytosis and extracellular vesicles, receiving nutrients, small non-coding RNAs (sncRNAs), proteins, metabolites, hormones, and other signaling molecules (Bline et al., 2020). The Sertoli cell barrier (also known as the blood-testis barrier) does not protect the Sertoli cells or SSCs from toxicants, but rather helps to protect the meiotic (spermatocytes) and haploid (spermatids) male germ cells, particularly from immunological response. Also, it appears this barrier does not exist at birth, infancy, or youth; it develops in the early phase of puberty, around 11 to 13 years of age (Mruk & Cheng, 2015).

During spermiogenesis, the final stage of spermatogenesis, haploid spermatids undergo profound changes in both the composition and the compaction state of their nuclear chromatin. When round spermatids differentiate into elongating spermatids, histones are first replaced by transition proteins, and then by protamines, which condense the chromatin into the minute head of the spermatozoa while also offering protection of the paternal genome from potential damage caused by adverse factors (e.g., free radicals) (Wouters-Tyrou et al., 1998). However, about 5–10% of histones persist in human sperm, including within promoter regions of genes enriched for developmental, neuronal and metabolic pathways (Lempradl, 2020). Therefore, these retained histones and their covalent modifications, in combination with DNA methylation, are, in principle, candidates to carry epigenetic information between generations (Kremsky & Corces, 2020; Martini et al., 2020).

### Considerations for Female Germline Development

Primordial germ cells, once arrived at the female genital ridge, become oogonia, which then multiply themselves via mitosis before entering meiosis between week 10 to 20 (Kurimoto & Saitou, 2019). Soon after the initiation of meiosis, oocytes become arrested at the diplotene stage of the first meiotic prophase in the third trimester (Sanchez & Smitz, 2012). Unlike male germ cells, the female germ cells remain globally demethylated for a lengthy period of time, until puberty, when remethylation occurs during the course of folliculogenesis. While CpG methylation is largely established in oocytes of the germinal vesicle stage, non-CpG DNA methylation continues to accumulate as oocytes mature into metaphase II (Yu et al., 2017). Overall, the oocyte DNA methylation patterns are distinct from those in sperm and somatic cells.

Similar to the male germ cells, the oocytes engage in continuous interactions with their somatic support cells, namely the granulosa cells, which provide nutrients, sncRNAs, proteins, metabolites, hormones, and other signaling molecules (Bline et al., 2020). Although there exists a blood-follicle barrier in the ovary, it is not ironclad, and chemicals that can pass the germ cell barrier include lipid-soluble compounds and the PPARγ agonist rosiglitazone (Janesick & Blumberg, 2016). Studies have shown, for example, that anesthetic chemicals, which are lipid-soluble, are retained in follicular fluid following patient anesthesia (Christiaens et al., 1999), that excessive fluoride damaged oocytes in a mouse model (Wang et al., 2017), and that the halogenated anesthetic gas isoflurane resulted in a reduced number of developing follicles and an increased number of atretic follicles in mice (Tang et al., 2020). Around 300,000 oocytes remain at the start of puberty and there is a continued loss of about 1,000 follicles per month that accelerates with age, and reductions in the ovarian reserve are permanent, as female germ cells cannot be replaced (Orsi et al., 2014). Data from experimental animal models and epidemiological studies indicate that exogenous chemicals can contribute to reduction in the ovarian reserve (Ge et al., 2019).

### Pre-implantation Reprogramming

One challenge to the idea of environmental exposures that affect the epigenome of the germline having any significant impact on the subsequent offspring is the wave of epigenetic reprogramming that takes place in the preimplantation embryo, which erases much of the epigenetic information, including histone modifications and DNA methylation which had been established in the egg and sperm during gametogenesis. This process, while robust, is incomplete as thousands of regions escape reprogramming and imprinted sites remain protected from reprogramming events (Seisenberger et al., 2013). Some loci associated with metabolic and neurological disorders are resistant to DNA demethylation, revealing potential for intergenerational epigenetic inheritance that may have phenotypic consequences (Tang et al., 2015). Therefore, alterations in DNA methylation induced by environmental factors during the course of gametogenesis may be retained if they are present at genomic sites that resist reprograming after fertilization (Schrott & Murphy, 2020). However, even sites that become demethylated in the embryo may be able to maintain the memory of their germline methylated state if bound TFs in the gametes help guide the remethylation process in the post-implantation embryo (Kremsky & Corces, 2020). In addition to chromatin-based epigenetic content being passed directly from the gametes to the zygote, there is also the possibility that non-coding RNAs expressed in the germline can provide a source of heritable information between generations (Gapp et al., 2020).

### De Novo Germline Mutation

Although we focus here on the epigenome, it is also possible that toxicants can act as germline mutagens, perhaps accounting for de novo germline mutations as well as somatic mosaic mutations in early embryonic development that correlate with ASD phenotypes (Pugsley et al., 2021). With respect to de novo germline mutagenesis, germ cells generally have a strong ability to cope with DNA damage (García-Rodríguez et al., 2018), but aging primary oocytes may exhibit a decreasing ability to repair DNA damage during the lengthy postnatal period of meiotic arrest (Myers & Hutt, 2013). Mature spermatozoa have a limited capacity to perform DNA repair and are unable to complete apoptosis, which can result in the retention of spermatozoa with damaged and/or fragmented DNA (García-Rodríguez et al., 2018). Accumulated DNA damage can result in genetic abnormalities in the offspring (Marchetti & Wyrobek, 2005). In addition, destabilizing germline insults such as benzo(a)pyrene, a mutagenetic component of tobacco smoke and air pollution, should be considered as potential factors that raise risk for somatic mosaicism in the offspring (Beal et al., 2019; Godschalk et al., 2015).

## Evidence of Germ Cell Alterations Caused by Exogenous Factors in Mammalian Models and Human Studies

We will briefly discuss four categories of substances for which germline exposure has been linked to abnormal brain development and/or behavior in offspring: halogenated anesthetic gases; hormone-disrupting exposures; products related to smoking; and valproic acid. This is not intended as an exhaustive list, but merely to illustrate the principle with actual exposures that are common in the human population and that to some extent have been investigated for heritable impacts on offspring neurodevelopment.

### Halogenated General Anesthetic Gases

Based on studies to date, the toxicants that are perhaps of paramount concern are halogenated anesthetic gases. The first of these gases, halothane, was introduced into practice in 1956, followed by many others including enflurane (1972), isoflurane (1981), desflurane (1992), and sevoflurane (1995) (Whalen et al., 2005). The rate of surgical procedures in the U.S. has been increasing annually. In 2006, an estimated 53.3 million surgical and nonsurgical procedures were performed in U.S. ambulatory surgery centers, and in 2010, 51.4 million inpatient procedures were performed in nonfederal hospitals in the U.S. (Forum, 2017). The number of surgeries performed globally has rapidly increased, from 226.4 million in 2004 to 312.9 million in 2012, according to World Health Organization estimates (Weiser et al., 2016). The most commonly used inhaled anesthetics in these procedures are nitrous oxide and the halogenated gases, which are typically administered in combination with intravenous anesthetic agents such as midazolam or propofol (Clar et al., 2021).

The halogenated anesthetic agents commonly employed in surgical procedures are small, potent, lipophilic molecules that diffuse through vessel-rich tissues, including the gonads, with the clinical purpose to interrupt nerve signals and induce a global suppression of the nervous system. They may be used in unusually high concentrations in young children owing the immaturity of their GABAergic system (Li et al., 2019). Anesthetic gases can cause significant DNA damage (Schifilliti et al., 2011), changes in gene expression, and epigenetic alterations (Martynyuk et al., 2020; Wang et al., 2021), in both exposed somatic and germ cells (Escher & Ford, 2020; Kaymak et al., 2012; Martynyuk et al., 2020; Wang et al., 2021). The damage caused by the gases also manifests in morphological and functional impairments in sperm (Coate et al., 1979; Land et al., 1981; Tang et al., 2020; Wang et al., 2008). The gases are also widely observed to act as steroid hormone disruptors, inducing dysfunction in the gonadal tissues and cells, with adverse impacts on germ cell integrity (Arena & Pereira, 2002; Kaya et al., 2013; Xu et al., 2012).

Studies in rodent models have repeatedly demonstrated that germline exposure to halogenated anesthetic gases can exert adverse brain and neurobehavioral outcomes in live-born progeny. The first of these dates back to 1981, a small study finding maternal line F2 generation of halothane-exposed gestating F0 female mice to be “significantly slower than control mice throughout the training” on all days of testing and all configurations of a maze test. Specifically, in a maze test used to assess learning, control mice made significant progress in all maze settings by the third training period. In contrast, F2 mice, born to the F1 females exposed to halothane in utero took until the seventh training period to learn the maze. The authors concluded that the impaired learning in the F2 “suggests that the anesthetic agent may have caused a genetic aberration” in the exposed mothers’ fetal eggs (Chalon et al., 1981). In a 1984 paper, the same lab reported that enflurane caused impaired learning function in the generation born of the exposed germ cells, this time later-stage sperm instead of early-stage eggs (Tang et al., 1984). The researchers remarked that it “seems likely that spermatogenetic changes, caused by enflurane, are associated with genetic alterations” that affected the pups’ brain development. After these papers raised the specter of potential adverse heritable impacts of general anesthesia, this important question for public health seemed to fall into the abyss, and more than three decades passed before another paper was published on this topic.

In the past few years several studies have revisited this question and have reached similar conclusions, while adding the dimension of implicating epigenetic mechanisms. In the first of these studies, sub-clinical concentrations of sevoflurane (2.1% sevoflurane for 6 h) were administered to male and female neonate rat pups and the directly exposed F0 animals and their F1 progeny were examined (Ju et al., 2018). Using the elevated plus maze and the Morris water maze tests, it was found that the F0 and F1 male animals exhibit abnormal behaviors in both tests, indicating increase in anxiety and impairment in spatial memory. These behavioral abnormalities were associated with changes in gene expression of the potassium chloride cotransporter 2 (*Kcc2*). *Kcc2* expression is reduced by 20–40% in the hypothalamus and less than 20% in the hippocampus of F0 and F1 male animals compared to unexposed controls (Ju et al., 2018). DNA methylation in the promoter of the *Kcc2* gene was examined in sperm of F0 males and hypothalamus and hippocampus of F1 males, and found to increase significantly in the six CpG sites examined after sevoflurane exposure. These data suggest that the down-regulation of *Kcc2* gene expression and increased promoter CpG methylation in the F1 hypothalamus is associated with the increased in *Kcc2* promoter CpG methylation of the F0 sperm. *Kcc2* is a central nervous system (CNS) neuron-specific chloride potassium symporter localized at excitatory synapses that is essential for synaptic inhibitions, synaptic spin morphogenesis and neuroplasticity. Mutations or changes in *Kcc2* expression are involved in many neurological diseases including brain trauma, epilepsies, autism and schizophrenia (Agez et al., 2017). These findings suggest that sevoflurane could induce a nongenetic effect in early-stage germ cells, causing some sex-specific brain and behavioral abnormalities in the next generation, even when used at low concentrations. An editorial accompanying the paper reporting these results noted that general anesthetics may modulate developmental neuroplasticity in the next generation via changes in gene expression and DNA methylation (Vutskits et al., 2018).

Studies by the same group found that expression of DNA methyltransferase 3a and 3b (*Dnmt3a* and *Dnmt3b*, enzymes that catalyze the transfer of a methyl group to DNA) in the hypothalamus of F1 animals was increased by more than 40% compared to unexposed control males (Xu et al., 2020). When the animals were treated with Decitabine, a methyltransferase inhibitor, prior to sevoflurane exposure, the expression of *Dnmt3a*, *Dnmt3b*, and *Kcc2* in the hypothalamus of F1 animals were similar as unexposed control animals and the animals exhibit normal behaviors. These data suggest that DNA methyltransferase activity might be involved in the response to sevoflurane exposure at the *Kcc2* locus. In the future, it would be interesting to perform genome-wide analyses of changes in gene expression and DNA methylation in this system.

In addition, administration of sevoflurane to young adult rats (with more mature germ cells) resulted in similar, though not identical, abnormalities in parental germ cells and in male offspring of exposed sires and dams (Ju et al., 2019). Notably, the lab’s experiments suggested that compared to the somatic cells, the germ cells are more sensitive to the deleterious effects of sevoflurane, raising the possibility that male offspring may be affected even when the anesthesia level/duration is insufficient to induce significant abnormalities in exposed parents (Martynyuk et al., 2020).

Another lab recently performed experiments with some similar aims but looking only at the offspring brain as an endpoint rather than the parental germ cells or offspring behaviors. After exposing neonatal female rats to sevoflurane, they bred the females and found their F1 offspring’s brains exhibited epigenetic abnormalities, including reduced DNA methylation in hippocampal neurons and upregulation of *Arc* and *Junb* mRNA expression in F1 males born to F0 exposed females, an effect linked to functional decline in learning and memory. This effect was sexually dimorphic, again only noted in the F1 male progeny (Chastain-Potts et al., 2020).

A recent study from another lab demonstrated the molecular basis for neurodevelopmental pathology in offspring of sperm of F1 sons of pregnant mice exposed to sevoflurane (Wang et al., 2021). Gestating F0 mice were exposed at day E12.5 of F1 embryonic development for 2 h, as this is the time when the germline of the exposed fetus is fully demethylated and may be more susceptible to environmental exposures. Adverse behavioral defects were observed in more than 38% of the directly exposed F1 males, including sociability deficits and increased anxiety as measured by the three-chamber sociability test, bedding shredding and marble burying tests. By outcrossing the F1 males to unexposed females for two generations, sevoflurane was found to have both “intergenerational” (F2 derived from exposed germline) and “transgenerational” (F3 derived from germline never exposed to sevoflurane) actions. In fact, 44–47% of the F2 and F3 showed the same behavioral problems as the F1 males (females were not tested). Based on preliminary data from one of our labs (VGC), these behavioral phenotypes correlate with reduced neonatal brain size and weight. However, the brain size and weight differences were not apparent in the mature adult mice. The inter- and transgenerational inheritance through the male germ cells was confirmed by Assay for Transposase-Accessible Chromatin sequencing (ATAC-seq) experiments in sperm of the F1 and F2 generations, which showed a dramatic recruitment of TFs to enhancer sequences of genes found to be associated with ASD, including* Arid1b*, *Ntrk2*, and *Stmn2* (Wang et al., 2021). These results and the ones described above point to a correlation between exposure of laboratory animals to sevoflurane, alterations of the transcriptional landscape in the germline, changes in progeny’s neural cell epigenomes, and the development of behavioral phenotypes similar to those displayed by humans diagnosed with autism. Therefore, evidence obtained in mouse models by independent laboratories suggest that sevoflurane, one of the most commonly utilized GA agents in surgery, could led to heritable alterations in the epigenome of the germ cells and brain, through changes in DNA modifications, gene expression and transcription factor occupancy.

In human cohorts, research on germ cell impacts of general anesthesia has been surprisingly sparse, but intriguingly two studies point to significant molecular vulnerabilities. In terms of the epigenome, one study examining obesity and bariatric surgery found significant changes in spermatozoa DNA methylation in 1509 genes approximately one week after surgery, with persistent effects in 1004 genes and 1116 CpG positions a year later (Donkin et al., 2016). Though the authors attributed these changes to weight loss and nutritional factors, the sudden nature of the effects point to the anesthesia as possibly the more salient exposure (Martynyuk et al., 2020). A 2012 study on DNA damage in sperm in vitro after exposure to various concentrations of halothane, isoflurane, desflurane and sevoflurane was conducted by the classic DNA damage “comet” assay (Kaymak et al., 2012), which assesses DNA damage via single cell gel electrophoresis (Azqueta & Collins, 2013). The genotoxic effect was dose-dependent for isoflurane and sevoflurane, and halothane was most strongly genotoxic, but this effect was not dose dependent. No genotoxic effect was observed for desflurane. The study was preliminary in nature, however, offering no data on repeated exposures or different durations (Kaymak et al., 2012). It is also worth noting that a recent epidemiological study on a large Danish cohort found a two-fold higher autism risk in offspring of parents who had been born very preterm, that is, less than 32 weeks of gestation (Xiao et al., 2021). Although this was not part of the study’s evaluation, it is well known that premature infants, and in particular very preterm infants, undergo sharply higher rates of early life drug exposure, including anesthesia for surgery, opiates, oxygen, and corticosteroids (Smrcek et al., 2005).

Taken together, the studies offer evidence that agents of general anesthesia can induce molecular changes in germline, changing transcription of key brain development genes and inducing adverse neurodevelopmental outcomes in progeny, particularly males.

### Synthetic Steroids, Endocrine-Disrupting Chemicals and Endocrine Disease

In recent decades, humans have been increasingly treated with synthetic hormone drugs and exposed to many environmental substances that act as EDCs (Diamanti-Kandarakis et al., 2009). Most of these substances affect molecular signaling through the superfamily of nuclear receptors, which act as DNA-binding TFs with powerful capabilities of modifying the epigenetic landscape and gene expression programs (Ozgyin et al., 2015). Numerous studies support the hypothesis that alterations in endocrine systems influence the epigenetic information of the germline which may lead to neurodevelopmental and behavioral abnormalities in subsequent generations.

Several studies have investigated heritable impacts of synthetic steroid drugs. In a guinea pig model, F0 gestational treatment with a clinically relevant dose of the synthetic glucocorticoid betamethasone led to abnormalities in the F2 generation, including modified physiology of the hypothalamic–pituitary–adrenal (HPA) axis and increased locomotor activity in a novel location (Moisiadis et al., 2017). In a mouse study, elevated paternal glucocorticoid exposure altered the profile of small noncoding RNA profile in sperm and resulted in increased anxiety-like behavior in next-generation (F1) males, but decreased the same behaviors in F2 male and female offspring. In F2 males only there was evidence of enhanced depression-like behaviors (Short et al., 2016). In humans, grandchildren of pregnant women administered the notorious synthetic estrogen diethylstilbestrol (DES) (data from descendants of over 47,000 DES-treated women) exhibit significantly increased risk for ADHD through the maternal line (Kioumourtzoglou et al., 2018).

In addition to endocrine alterations due to exogenous administration of drugs, aberrant status of endogenous hormones may also impact germline and influence neurological phenotypes in subsequent generations. This may occur due to chronic stress, which elevates circulating levels of glucocorticoids. Neonatal, juvenile and adult stress may change the profile of microRNAs, a category of sncRNAs, in the sperm and lead to aberrant programming of the HPA axis and to anxiety and other neurological phenotypes in subsequent generations (Dickson et al., 2018; Gapp et al., 2014; Jawaid et al., 2018; Manners et al., 2019; Morgan & Bale, 2011; Rodgers et al., 2013, 2015; Saavedra-Rodríguez & Feig, 2013). Alterations in thyroid hormone, which occurs in women with thyroid disease, can also cause intergenerational effects, affecting neuroendocrine function (Anselmo et al., 2019; Bakke et al., 1977). Furthermore, sperm epigenetic information is altered in a mouse model of developmental overexposure to thyroid hormone (Martinez et al., 2020). This exposure causes hypomethylation in the promoter of genes involved in brain development that are also implicated in ASD and other neurological disorders. F2 generation descendants of exposed male and female mice exhibit altered neonatal brain gene expression programs and abnormal behaviors (Martinez et al., 2020).

Studies involving environmental EDCs have also found links between germline exposure and abnormal neurobehavioral outcomes in the offspring. Exposure of F1 fetal rats to the androgenic fungicide vinclozolin or to polychlorinated biphenyls, which mimic the structure of thyroid hormones, led to socio-sexual behavioral abnormalities in the F2 progeny, with males most affected (Krishnan et al., 2018, 2019). This was associated with abnormal expression of steroid hormone receptors (estrogen receptor α, androgen and progesterone receptors) in the medial preoptic area and ventromedial nucleus of the hypothalamus (Krishnan et al., 2018, 2019). Several studies have examined the effects of bisphenol A (BPA), a compound with estrogenic properties, on social behaviors in mice of the first and subsequent generations (Goldsby et al., 2017; Wolstenholme et al., 2012, 2019). Mice exposed to BPA in utero exhibited reduced social interest compared to control mice, but sociability was increased in subsequent generations (Wolstenholme et al., 2012). The brains of BPA-exposed fetal mice exhibited reduced expression of oxytocin and vasopressin, critical neuropeptides controlling social behaviors in mice and humans which have been implicated in ASD and schizophrenia. The brain expression of vasopressin and estrogen receptor α, which regulates the expression of oxytocin (Young et al., 1998) and vasopressin (Scordalakes & Rissman, 2004) was also reduced in BPA mice (Wolstenholme et al., 2012). The decrease in vasopressin expression persisted until the F3 generation in the BPA lineage, which also exhibited severe deficits in social recognition and the expression of postsynaptic density genes (Wolstenholme et al., 2019). Interestingly, BPA-line F3 generation mice also exhibited marked abnormalities in the expression of imprinted genes, especially the maternally expressed gene Meg3 (Drobna et al., 2018). It is worth noting that these changes in gene expression were found in areas related to the sexual differentiation of the brain, including the lateral septum, amygdala, preoptic area, hypothalamus and bed nucleus of the stria terminalis (Drobna et al., 2018; Goldsby et al., 2017).

Mechanistically, EDCs may behave similarly to the endogenous hormones that they mimic and bind to or interfere with the binding of endogenous hormone receptors (e.g., steroid receptors) or other binding proteins involved in hormone physiology and action, ultimately impacting receptor chromatin modification and transcriptional functions (Lakshmanan & Shaheer, 2020; Martini et al., 2020). Directly, by binding to hormone receptors, or indirectly, by changing recruitment patterns of TFs, including Ctcf, EDCs could reprogram the germline at different stages of development (Fiorito et al., 2016). DNA-bound TFs could then modify accessibility of epigenetic modifiers to specific genomic loci. For example, ATAC-seq experiments carried out with sperm from the F1 through F6 progeny of mice exposed to BPA in utero reveal disruptions at binding sites for Ctcf, Foxa1, Esr1 and Ar (Jung et al., 2020). The sperm disruptions persist (or lead to subsequent disruptions) after fertilization in somatic cells of the post-implantation embryo, affecting cell differentiation and development in the next generation, eliciting abnormal phenotypes in the adult organism. These abnormal patterns of transcription may affect genes critical for the development of neurological and endocrine functions in the offspring (Martini et al., 2020). This has been shown to be the case for BPA-induced alterations in sperm in the binding of Ctcf to an enhancer of the *Fto* gene. These alterations are maintained in the hypothalamus and affect the differentiation of POMC and AgRP neurons in the arcuate nucleus, leading to increased food consumption and obesity (Jung et al., 2020).

Given the dramatic surge in the medical use of synthetic hormones and environmental exposure to EDCs over the course of the past six decades, it is possible that some of these exposures are altering the transcriptional program of germline, conferring risk for dysregulated brain development and abnormal behaviors.

### Tobacco and Related Products

Whether germline exposure to tobacco, its metabolites, or related products can influence autism risk may depend on timing and dose. While maternal smoking either before or during pregnancy may be associated with a variety of risks to the fetus, evidence for an increase in autism risk is low (Lee et al., 2012; Rosen et al., 2015), with perhaps only a slightly elevated risk when the mother was a heavy smoker (von Ehrenstein et al., 2020). However, in contrast to earlier studies, a recent epidemiological study based on two large cohorts in Korea found paternal smoking correlated to an increased likelihood of ASD in offspring. The authors concluded that elimination of paternal smoking might reduce the risk of having a child with ASD by as much as 11–14% (Kim et al., 2021). Fetal germline impacts were the subject of study in the Avon Longitudinal Study of Parents and Children (ALSPAC) cohort, which linked grandmaternal smoking in pregnancy with an increased risk for autism traits and diagnosed autism in grand-offspring through the maternal line (Golding et al., 2017), though it lacked data on dose effects.

Both mutagenic and epimutagenic factors may be at play. Paternal smoking affects the mutation rates in sperm (Axelsson et al., 2018; Haervig et al., 2020), for example by increasing DNA adducts caused by a metabolite of benzo(a)pyrene (BaP), a known carcinogen and main component of tobacco smoke (Beal et al., 2019; Laubenthal et al., 2012; Linschooten et al., 2013). Studies in rodents demonstrate that ingestion of nicotine by gestating dams increased risk for ADHD-like behaviors in the F2 generation (Buck et al., 2019; Zhu et al., 2014), with epigenetic mechanisms in the exposed germline being implicated (Buck et al., 2019). More recently, a study showed that mouse sires exposed to nicotine and saccharin, a mixture common in vaping products, produced male (females were not examined) offspring with elevated activity and reduced spatial memory (McCarthy et al., 2020). Both nicotine and saccharine exposure produces significant changes in DNA methylation at promoter regions of dopamine receptor genes in spermatozoa, suggesting that epigenetic modification of sperm DNA may link the exposure to the behavioral phenotypes (McCarthy et al., 2020).

The generational effects of cannabis use have also emerged as a concern for heritable neurobehavioral effects. One study has reported alterations in DNA methylation in human sperm in men that were frequent cannabis users as compared with non-smokers (Murphy et al., 2018). A follow-up study looked at the effects of cannabis exposure on DNA methylation of the gene Disks-large associated protein 2 (*DLGAP2*), which is implicated in ASD (Schrott et al., 2020). This gene exhibited 17 differentially methylated CpG sites by Reduced representation bisulfite sequencing (RRBS) in the sperm of cannabis-exposed men compared to controls. In the brains of rats born to THC-exposed fathers, significant loss of methylation was detected at the same CpG sites in the nucleus accumbens as in the sperm of the exposed fathers, suggesting paternal exposure could alter the epigenetic profile of the offspring.

### Valproic Acid

Histone deacetylase (HDAC) inhibitors are drugs commonly used to treat seizure disorders and other mental conditions that act directly on the epigenome, by inhibiting removal of the acetyl groups from lysine residues on histones, leading to the establishment of a transcriptionally silenced chromatin. Fetal exposure to the HDAC inhibitor valproic acid (VPA) induces an autism-like neurobehavioral phenotype in mice and is known to cause autism-related neurobehavioral impairment in humans (Roullet et al., 2013). Evidence from rodent models is also emerging that VPA causes neurobehavioral impacts on the next generation via an exposed germline. VPA induced epigenetic inheritance of an autism-like phenotype in mice through the paternal germline in the first and second generation (Choi et al., 2016). Similarly, paternal VPA exposure increased behavioral abnormalities in adult offspring, with increased levels of acetylated histone H3 in the testicular tubules of sires**,** but, surprisingly, decreased the levels of acetylated histone H3 in the brain of adult offspring (Ibi et al., 2019). In this case, the relationship between effects in the germline and somatic tissues would have to be indirect. In another mouse study on a fetal germline exposure to VPA, the offspring exhibited early behavioral alterations and increased expression of endogenous retroviruses (Tartaglione et al., 2019).

To our knowledge, no human study has investigated outcomes after fetal germline exposure to HDAC inhibitors. However, paternal use of antiepileptic medications (which are often HDAC inhibitors), prior to conception tend not to be associated with an increased risk of autism in offspring (Tomson et al., 2020). Earlier stage exposures remain to be investigated.

## Implications for Autism Research

Here we have described how non-genetic heritable information induced in the germline by exposure to exogenous toxicants and other environmental factors could contribute to the etiology of neurodevelopmental and behavioral abnormalities, some of relevance to ASD. While the field largely focuses on genetic architecture, we instead stress the importance of an epigenetic landscape that may be perturbed by molecular events in the parental germ cells. This paradigm has many implications for future research directions.

### Mammalian Models

Mammalian models can be employed to look at exposures to both male and female germline with various agents, mixtures, and intensities across the span of germ cell developmental windows. Endpoints may include impacts on germline genetics and epigenetics and neurological phenotypes in the offspring and descendants, including brain gene transcription and epigenetic profiles in brain, neuromorphology and cytoarchitecture and behavior. Agents of interest may include those reviewed above, but there may be many other factors capable of exerting intergenerational effects. In this regard, some medications given to women during pregnancy (for example, anti-nausea drugs, anti-preterm birth steroids, psychoactive drugs), especially those with known mutagenic or epigenetic properties, such as chemotherapeutic agents, should raise concern (Kaplanis et al., 2021). A similar concern applies to an increasingly long list of environmental chemicals that act as endocrine disruptors which could affect the germline directly, or indirectly via altering endogenous endocrine systems. Other exposures may derive from specific occupational hazards in subsets of the population. For example, it has been observed that maternal occupational exposure to solvents may increase the risk for ASD in the offspring (McCanlies et al., 2019).

### Epidemiology

Although prospective human studies would be difficult, retrospective studies should be feasible in many available cohorts with reliable data about specific exposures (for example, surgery histories of the parents, medications dispensed to women during pregnancy, medication history of the fathers) and neurodevelopmental outcomes in offspring of the exposed germ cells. Additionally, in light of a machine-learning based study from Denmark finding that family history of multiple mental and non-mental conditions can identify more individuals at highest risk for ASD than only considering the immediate family history of ASD (Ejlskov et al., 2021), further big data approaches focusing on specific exposures such as surgery, that are associated with health conditions, warrant further investigation. While register-based studies from the Nordic countries may be the most suitable for epidemiological studies involving more than one generation, other specific cohorts in other countries may also be possible, as has already been seen with ALSPAC (Golding et al., 2017), and Nurses Health Study II (Kioumourtzoglou et al., 2018). Epidemiologic approaches for performing intergenerational associations were recently explored in McGee et al. (2020).

### Sex Bias of Autism

Studies in mammalian models often show that males born of toxicant-exposed germ cells at increased risk for brain and/or neurobehavioral abnormality, as compared to females (Chastain-Potts et al., 2020; Ju et al., 2018, 2019; Krishnan et al., 2018), although the underlying mechanisms for this sex bias have not been ascertained. Given the sex steroid-like properties of many of the drugs and chemicals discussed in this paper, epigenetic programming of sex steroid target genes, including those regulating the HPA-gonadal axis and sex hormone output, may occur. This in turn may impact processes of brain maturation in a sex-specific manner, ultimately resulting in sexually dimorphic neurobehavioral outcomes. Sexual dimorphism in intergenerational effects may also be the result of altered epigenetic information affecting the X chromosome; due to X chromosome inactivation in females, cell mosaicism may result in less severe and more variable impacts. However, these and other questions remain to be investigated.

### Broader Autism Phenotype (BAP)

The BAP, a collection of sub-diagnostic traits sometimes seen in parents of children with autism (Rubenstein & Chawla, 2018) could in some cases be accounted for by that parent’s developmental exposures, for example in utero exposure to synthetic sex steroids, or early life exposure to high doses of general anesthesia, as both exposures are associated with neurobehavioral difference and impairments (Ing et al., 2021; Reinisch & Karow, 1977; Warner et al., 2018). Indeed, there is some early evidence that perinatal exposure to general anesthesia administered as part of a C-section, may increase risk for autism in the exposed individual (Huberman Samuel et al., 2019; Yang et al., 2021).

### Case Studies of Idiopathic Multiplex Families

The autism family population represents a vast and generally untapped resource for addressing novel questions about germline exposures. In particular, parents who have strong recurrence of autism in their offspring could be questioned about their (and therefore their germ cells’) exposure histories or that of their parents (Escher, 2021). While such data would be anecdotal in nature, it could contribute valuable information to generate new hypotheses about the heritable origins of autism. Similarly, the phenotypic heterogeneity of autism, seen even within families, may be explained by differences in molecular impacts within the pool of affected parental germ cells.

### Studies of Sperm of Exposed Males

Considering the dramatic increase in the use of genotoxic and epigenotoxic drugs and chemicals, relatively little attention has been paid to how these exposures affect the qualities of human sperm. A small study found that DNA from the sperm of men whose children had early signs of autism shows distinct patterns of regulatory tags that could contribute to the condition (Feinberg et al., 2015). While the study did not explore paternal exposure history, it found 193 differentially methylated regions (DMRs) where the presence or absence of DNA methylation was statistically related to autism scores based on the Autism Observation Scale for Infants (AOSI) in their offspring. Interestingly, 24% of AOSI-associated DMRs are also found in the cerebellum of autistic individuals, lending support to the idea of causal relationship between epigenetic alterations in sperm and in adult tissues. Similarly, a small study on sperm of fathers who have children with ASD observed a set of 805 DMRs in sperm that could potentially act as a biomarker for paternal offspring autism susceptibility (Garrido et al., 2021). Evaluating the genetic and epigenetic signatures in the sperm of males against the backdrop of their known environmental exposures and lifetime clinical histories may yield provocative insights currently unexamined in the literature. Unfortunately, it is relatively difficult to directly study the eggs of exposed mothers except perhaps as part of assisted reproduction procedures, and even then, harvesting procedures involve the use of exogenous hormones and anesthetics which may influence oocyte content.

### Critical Reevaluation of the Current Literature

Another important undertaking, and one not requiring any clinical, experimental or epidemiological studies, would simply involve a critical reevaluation of the current autism literature to take into account this third dimension of risk. Studies warranting reevaluation include but are not limited to those involving sharply increasing prevalence across the past several decades, heritability modeling and heightened sibling risk, broader autism phenotype among family members, sex ratios among affected offspring, and even the sex-specific differential ASD clinical manifestations. Of course, the study of genetic transcriptional dysregulation of autism brains would also benefit from taking into account these non-genetic factors.

## Conclusion

Despite decades of diligent effort, autism’s etiology, increase in prevalence, heritability, sibling recurrence, sex bias, heterogeneity and basis in early transcriptional dysregulation remain largely unexplained. In order to accelerate progress with respect to all of these questions, we propose adding the phenomenon of germline disruption to the conventional approaches of genetics and somatic environmental exposures. Expanding our notions of pathogenesis may not only help explain many unsolved questions, it may also inform future directions for prevention, diagnosis and effective treatment.
